# High-performance neural population dynamics modeling enabled by scalable computational infrastructure

**DOI:** 10.21105/joss.05023

**Published:** 2023-03-21

**Authors:** Aashish N. Patel, Andrew R. Sedler, Jingya Huang, Chethan Pandarinath, Vikash Gilja

**Affiliations:** 1 Department of Electrical and Computer Engineering, University of California San Diego, United States of America; 2 Institute for Neural Computation, University of California San Diego, United States of America; 3 Center for Machine Learning, Georgia Institute of Technology, United States of America; 4 Department of Biomedical Engineering, Georgia Institute of Technology, United States of America; 5 Department of Neurosurgery, Emory University, United States of America; 6 These authors contributed equally

## Summary

Advances in neural interface technology are facilitating parallel, high-dimensional time series measurements of the brain in action. A powerful strategy for analyzing these measurements is to apply unsupervised learning techniques to uncover lower-dimensional latent dynamics that explain much of the variance in the high-dimensional measurements (Cunningham & Yu, 2014; Golub et al., 2018; Vyas et al., 2020). Latent factor analysis via dynamical systems (LFADS) (Pandarinath et al., 2018) provides a deep learning approach for extracting estimates of these latent dynamics from neural population data. The recently developed AutoLFADS framework (Keshtkaran et al., 2022) extends LFADS by using Population Based Training (PBT) (Jaderberg et al., 2017) to effectively and scalably tune model hyperparameters, a critical step for accurate modeling of neural population data. As hyperparameter sweeps are one of the most computationally demanding processes in model development, these workflows should be deployed in a computationally efficient and cost effective manner given the compute resources available (e.g., local, institutionally-supported, or commercial computing clusters). The initial implementation of AutoLFADS used the Ray library (Moritz et al., 2018) to enable support for specific local and commercial cloud workflows. We extend this support, by providing additional options for training AutoLFADS models using local clusters in a container-native approach (e.g., Docker, Podman), unmanaged compute clusters leveraging Ray, and managed compute clusters leveraging KubeFlow and Kubernetes orchestration.

As the neurosciences increasingly employ deep learning based models that require compute intensive hyperparameter optimization (Keshtkaran & Pandarinath, 2019; Willett et al., 2021; Yu et al., 2021), standardization and dissemination of computational methods becomes increasingly challenging. Although this work specifically provides implementations of AutoLFADS, the tooling provided demonstrates strategies for employing computation at scale while facilitating dissemination and reproducibility.

## Statement of need

**Figure 1:**
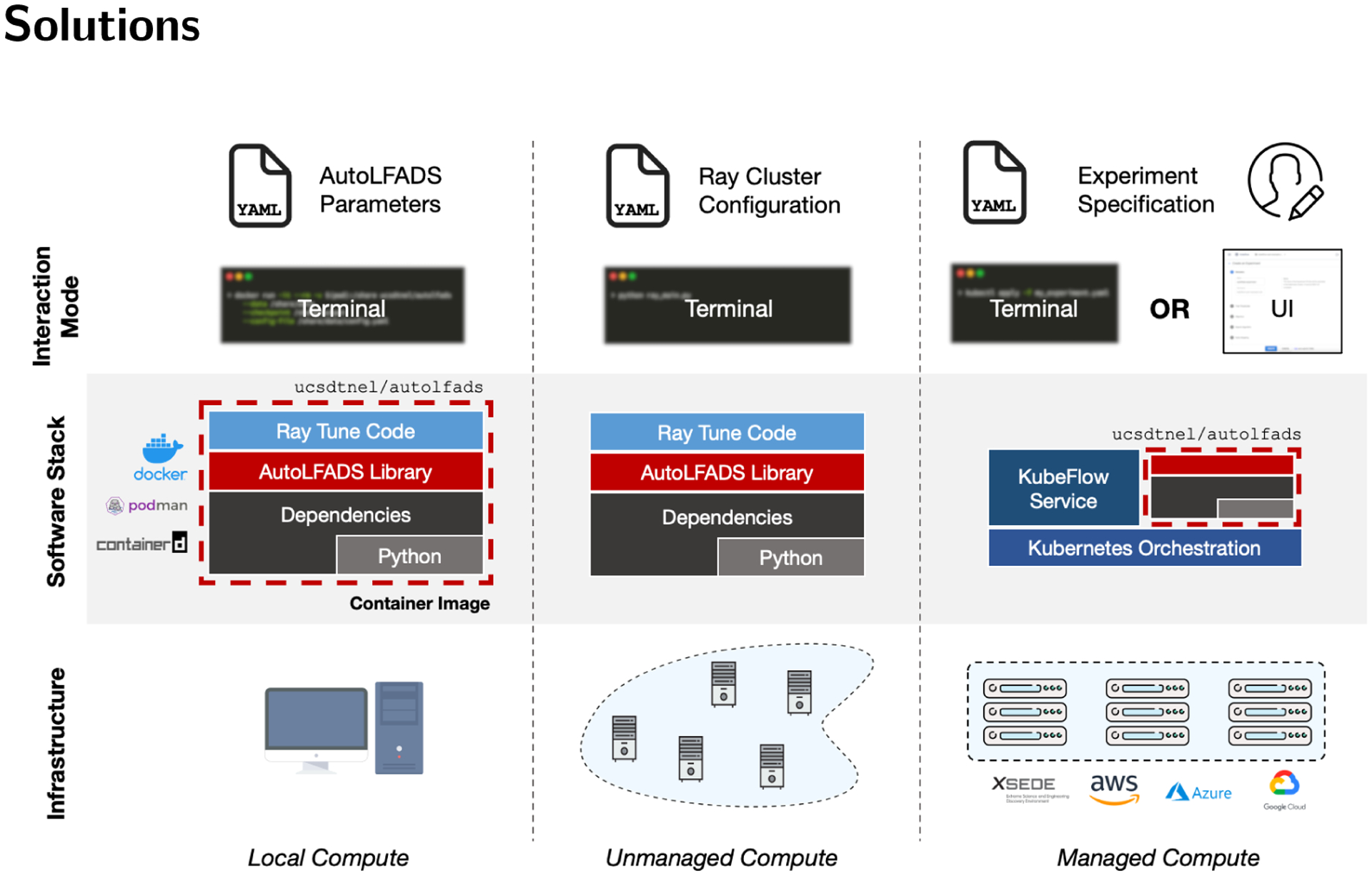
Solutions for running AutoLFADS on various compute infrastructures. (Left) This column depicts a local workflow: users leverage a container image that bundles all the AutoLFADS software dependencies and provides an entrypoint directly to the LFADS package. Container images are run with a supported container runtime (e.g., Docker, Podman, containerd). Users can interact with this workflow by providing YAML model configuration files and command line (CLI) arguments. (Middle) This column depicts a scalable solution using Ray: users start a Ray cluster by specifying the configuration (network location and authentication) as a YAML file. They install AutoLFADS locally or in a virtual environment, update YAML model configurations and hyperparameter sweep scripts, and then run the experiment code. (Right) This column depicts a scalable solution using KubeFlow: users provide an experiment specification that includes model configuration and hyperparameter sweep specifications either as a YAML file or using a code-less UI-based workflow. After experiment submission, the KubeFlow service spawns workers across the cluster that use the same container images as the local workflow.

Machine learning models enable neuroscience researchers to uncover new insights regarding the neural basis of perception, cognition, and behavior (Vu et al., 2018). However, models are often developed with hyperparameters tuned for a specific dataset, despite their intended generality. Application to new datasets requires computationally intensive hyperparameter searches for model tuning. Given the diversity of data across tasks, species, neural interface technologies, and brain regions, hyperparameter tuning is common and presents a significant barrier to evaluation and adoption of new algorithms. With the maturation of “AutoML” hyperparameter exploration libraries (HyperOpt, SkOpt, Ray), it is now easier to effectively search an extensive hyperparameter space. Solutions like KubeFlow (Kubeflow, 2018) additionally enable scaling on managed clusters and provide near codeless workflows for the entire machine learning lifecycle. This lifecycle typically begins with data ingest and initial evaluation of machine learning algorithms with respect to data, and then matures to compute intensive model training and tuning. Building upon these tools, we empower researchers with multiple deployment strategies for leveraging AutoLFADS on local compute, on ad-hoc or unmanaged compute, and on managed or cloud compute, as illustrated in Figure 1.

When training models on a novel dataset, it is often helpful to probe hyperparameters and investigate model performance locally prior to conducting a more exhaustive, automated hyperparameter search. This need can be met by installing the LFADS package locally or in a virtual environment. Isolating the workflow from local computational environments, we provide a pair of reference container images targeting CPU and GPU architectures. This allows users to treat the bundled algorithm as a portable executable for which they simply provide the input neural data and desired LFADS model configuration to initiate model training. This approach eliminates the need for users to configure their environments with compatible interpreters and dependencies. Instead, the user installs a container runtime engine (e.g., Docker, Podman), which are generally well-supported cross-platform tools, to run the image based solution. In addition to streamlining configuration, this approach enables reproducibility as the software environment employed for computation is fully defined and version controlled.

Scaling initial investigations may involve evaluating data on internal lab resources, which may comprise a set of loosely connected compute devices. In such a heterogeneous environment, we leverage Ray to efficiently create processing jobs. In this approach Ray spawns a set of workers on compute nodes that the primary spawner is then able to send jobs to. This approach requires users to provide a mapping of machine locations (e.g., IP, hostname) and access credentials. It provides useful flexibility beyond single node local compute, but requires users to manage compute cluster configuration details. Ray can also be deployed in managed compute environments, but similarly requires users to have knowledge of the underlying compute infrastructure configuration defined by the managed environment. In short, the Ray based solution requires researchers to specifically target, and potentially modify, compute cluster configuration.

To more effectively leverage large scale compute in managed infrastructure, such as those provided by commercial and academic cloud providers, we use KubeFlow which is a comprehensive machine learning solution designed to be operated as a service on top of Kubernetes based orchestration. This approach enables code-less workflows and provides a rich set of tooling around development (e.g., notebooks, algorithm exploration) and automation (e.g., Pipelines) that reduces research iteration time. In contrast to Ray, configuration requirements are algorithm focused and are generally agnostic to the lower level details related to compute cluster configuration. With this solution, Kubernetes manages the underlying compute resource pool and is able to efficiently schedule compute jobs. Within KubeFlow, we leverage Katib (George et al., 2020) – KubeFlow’s “AutoML” framework – to efficiently explore the hyperparameter space and specify individual sweeps. As KubeFlow is an industry-grade tool, many cloud providers offer KubeFlow as a service or provide supported pathways for deploying a KubeFlow cluster, facilitating replication and compute resource scaling.

The two distributed workflows provided, Ray and KubeFlow based, each have their respective advantages and disadvantages. The correct choice for a specific research end user is dependent upon their requirements and access to compute resources. Thus we provide an evaluation in Table 1 as a starting point in this decision making process.

## Evaluation

**Figure 2:**
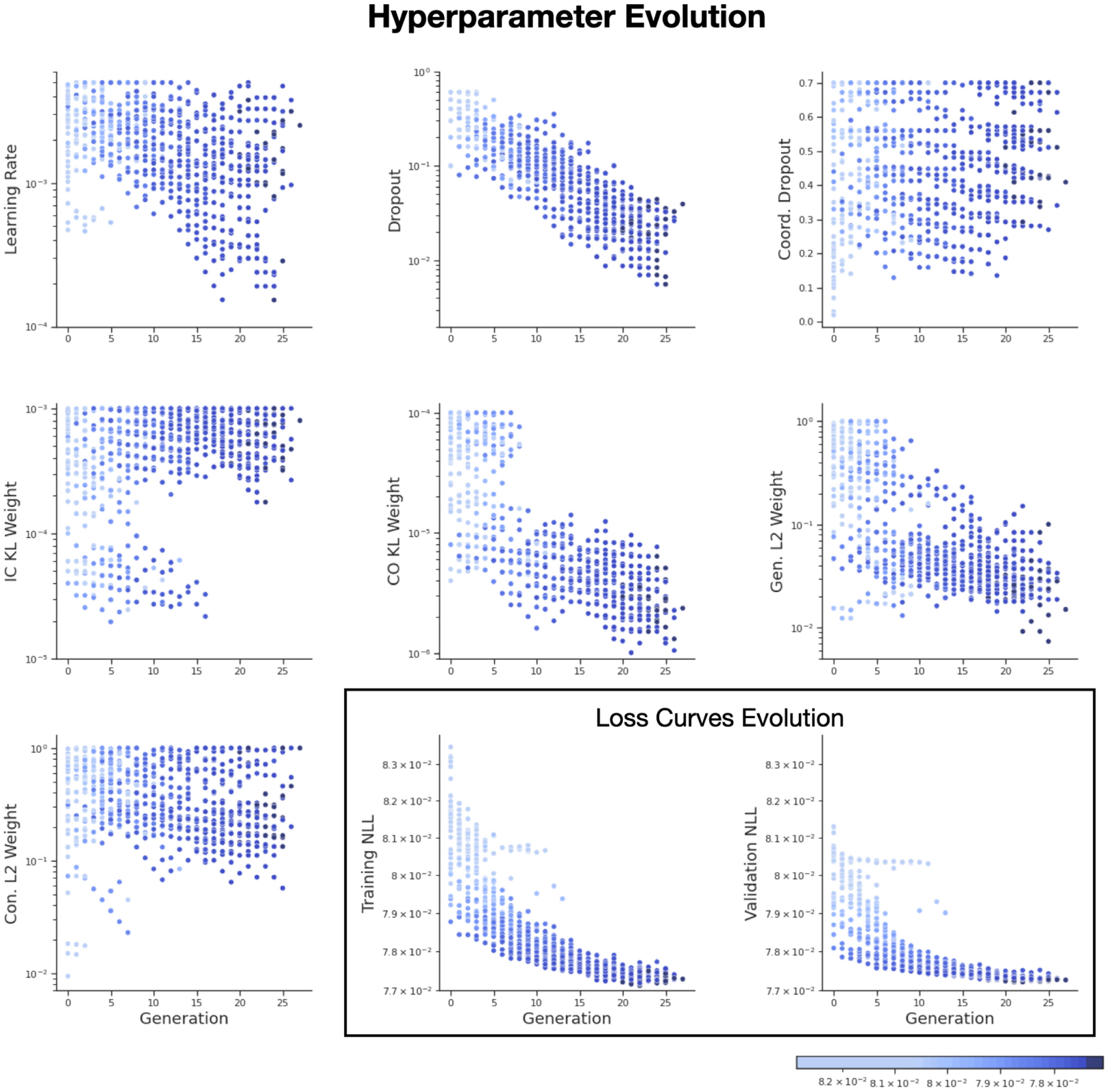
Evolution of loss curves and hyperparameters for AutoLFADS using KubeFlow. Each point represents each individual training trial. Points are colored by the exponentially-smoothed negative log-likelihood (NLL) loss at the end of each generation.

**Figure 3:**
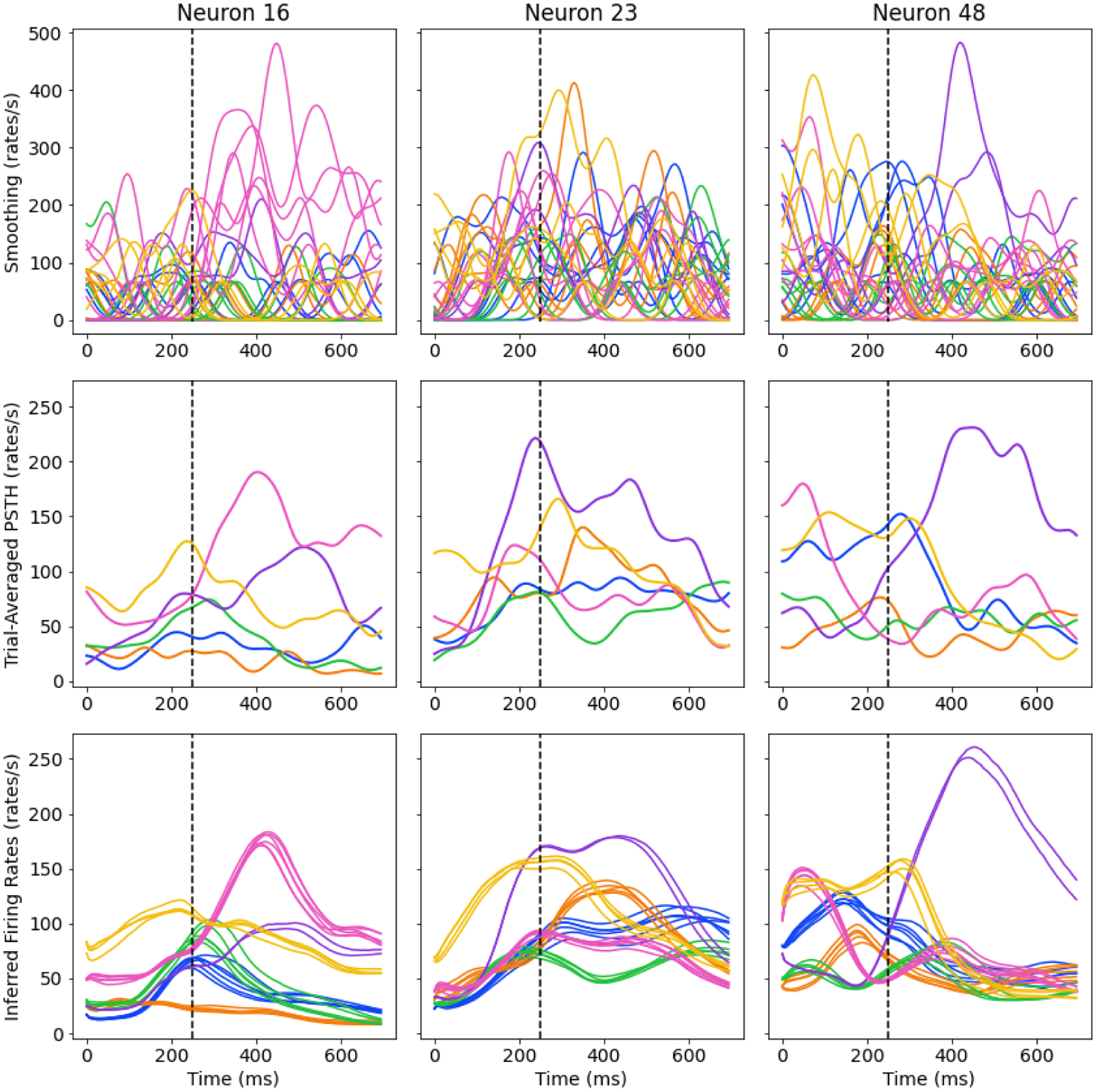
AutoLFADS inferred firing rates, relative to conventional estimation strategies, aligned to movement onset time (dashed vertical line at 250ms) for 3 example neurons (columns) and 6 example conditions (colors; out of 108 conditions). Smoothed spike rates calculated with a Gaussian kernel, 50 ms st.dev. (top), trial-averaged peristimulus time histogram (PSTH) (middle), and inferred firing rates of AutoLFADS on KubeFlow (bottom). The smoothed spikes and inferred firing rates are shown for corresponding trials in the validation set (trial count range from 2 to 6 per condition, shown as individual traces for each trial), while the PSTHs are calculated for all trials (trial counts range from 19 to 24 per condition, one trace per condition). In addition, the PSTHs are calculated based on the averaged spikes smoothed with a 70 ms st.dev Gaussian kernel, which corresponds to the definition of PSTHs used for the evaluation metric PSTH R2 (Table 1). In all subfigures, the inferred rates are calculated with a time resolution of 5ms.

A core innovation of AutoLFADS is the integration of PBT for hyperparameter exploration. As the underlying job scheduler and PBT implementation are unique in KubeFlow, we used the MC Maze dataset (Churchland & Kaufman, 2021) from the Neural Latents Benchmark (Pei et al., 2021) to train and evaluate two AutoLFADS models. One model was trained with the Ray solution and the other with the KubeFlow solution using matching PBT hyperparameters and model configurations to ensure that models of comparable quality can be learned across both solutions. A comprehensive description of the AutoLFADS algorithm and results applying the algorithm to neural data using Ray can be found in Keshtkaran et al. (2022). We demonstrate similar converged model performances on metrics relevant to the quality of inferred firing rates in Table 2 (Pei et al., 2021). In Figure 3, inferred firing rates from the KubeFlow trained AutoLFADS model are shown along with conventional firing rate estimation strategies. Qualitatively, these example inferences are similar to those described in Keshtkaran et al. (2022), showing similar consistency across trials and resemblance to peristimulus time histograms (PSTH). In Figure 2, we plot the hyperparameter and associated loss values for the KubeFlow based implementation of AutoLFADS to provide a visualization of the PBT based optimization process on these data. These results demonstrate that although PBT is stochastic, both the original Ray and novel KubeFlow implementations are converging to stable, comparable solutions.

## Figures and Tables

**Table 1:** Workflow overview. Summary of user burdens related to the three available deployment strategies.

Task	Local Solution	Unmanaged Solution	Managed Solution(this work)
Cluster management	Routine maintenance of software and hardware	*Same as Local Solution*	N/A
Cluster orchestration	N/A	Interacting with Ray clusters via CLI, manually setting up and tearing down clusters as needed Maintenance of YAML configuration files that specify network locations and access credentials for machines in the cluster	Interacting with Kubernetes clusters via user interfaces (GUI or CLI)
Dependency management	Running container images via Docker, Podman, or containerd	Installing dependencies in a virtual environment via Conda or pip	Specifying location of desired container image
Running distributed jobs	Developing YAML model configurations and running Python scripts that use Ray to sweep hyperparameters	*Same as Local Solution*	Providing model and hyperparameter sweep configurations to KubeFlow via YAML or code-less UI
Evaluating and tuning models	Visualizing loss curves and intermediate output in TensorBoard Flexible post-hoc analysis	*Same as Local Solution*	Visualizing loss curves and intermediate output in KubeFlow Flexible post-hoc analysis

**Table 2:** AutoLFADS Performance. An evaluation of AutoLFADS performance on Ray and KubeFlow. Test trial performance comparison on four neurally relevant metrics for evaluating latent variable models: co-smoothing on held-out neurons (co-bps), hand trajectory decoding on held-out neurons (vel R2), match to peristimulus time histogram (PSTH) on held-out neurons (psth R2), forward prediction on held-in neurons (fp-bps). The trained models converge with less than 5% difference between the frameworks on the above metrics. The percent difference is calculated with respect to the Ray framework.

Framework	co-bps (↑)	vel R2 (↑)	psth R2 (↑)	fp-bps (↑)
Ray	0.3364	0.9097	0.6360	0.2349
KubeFlow	0.35103	0.9099	0.6339	0.2405
Percent difference (%)	+4.35	+0.03	−0.33	+2.38
